# Detection of *ROS1* Gene Rearrangement in Lung Adenocarcinoma: Comparison of IHC, FISH and Real-Time RT-PCR

**DOI:** 10.1371/journal.pone.0120422

**Published:** 2015-03-05

**Authors:** Ling Shan, Fang Lian, Lei Guo, Tian Qiu, Yun Ling, Jianming Ying, Dongmei Lin

**Affiliations:** Department of Pathology, Cancer Institute & Hospital, Chinese Academy of Medical Sciences, Peking Union Medical College, Beijing, China; H. Lee Moffitt Cancer Center & Research Institute, UNITED STATES

## Abstract

**Aims:**

To compare fluorescence *in situ* hybridization (FISH), immunohistochemistry (IHC) and quantitative real-time reverse transcription-PCR (qRT-PCR) assays for detection of *ROS1* fusion in a large number of *ROS1*-positive lung adenocatcinoma (ADC) patients.

**Methods:**

Using IHC analysis, sixty lung ADCs including 16 cases with ROS1 protein expression and 44 cases without ROS1 expression were selected for this study. The *ROS1* fusion status was examined by FISH and qRT-PCR assay.

**Results:**

Among 60 cases, 16 (26.7%), 13 (21.7%) and 20 (33.3%) cases were *ROS1* positive revealed by IHC, FISH and qRT-PCR, respectively. Using FISH as a standard method for *ROS1* fusion detection, the sensitivity and specificity of IHC were 100% and 93.6%, respectively. Three IHC-positive cases, which showed FISH negative, were demonstrated with *ROS1* fusion by qRT-PCR analysis. The sensitivity and specificity of qRT-PCR for detection for *ROS1* fusion were 100% and 85.1%, respectively. The total concordance rate between IHC and qRT-PCR were 93.3%.

**Conclusion:**

IHC is a reliable and rapid screening tool in routine pathologic laboratories for the identification of suitable candidates for ROS1-targeted therapy. Some ROS1 IHC-positive but FISH-negative cases did harbor the translocation events and may benefit from crizotinib.

## Introduction

Lung cancer is the most frequent cause of cancer-related mortality worldwide [1]. Although the treatment has been improved with the use of platinum-based chemotherapy, the survival of patients with lung cancer remains poor [2]. Efforts have been made to identify driver oncogene mutation after the development of epidermal growth factor receptor (EGFR) tyrosine kinase inhibitors targeting EGFR in lung cancer. The echinoderm microtubule-associated protein-like 4—*ALK* (*EML4*-*ALK*) fusion was identified in 2% -7% of non-small cell lung cancer (NSCLC) patients [3–5]. An ALK inhibitor, Crizotinib, has been approved for the treatment of NSCLC patients with *ALK* gene rearrangement. ROS1 is a transmembrane tyrosine kinase receptor that has high homology with ALK in its protein kinase domain [6]. The *ROS1* rearrangement rendering a constitutively active tyrosine kinase was first discovered in NSCLC in 2007 and ~2% of NSCLC patients carried *ROS1* fusion [7]. The patients with *ROS1* fusion tend to be young never-smokers with adenocarcinoma, a population similar to those with *ALK*-rearranged NSCLC [8,9]. *ROS1* rearrangements rarely present simultaneously with *EGFR*, *KRAS* or *ALK* alterations [10]. Several fusion partners of *ROS1* have been identified, including *SLC34A2*, *CD74*, *KDELR2*, *GOPC (FIG)*, *TPM3*, *SDC4*, *LRIG3* and *EZR* [8,11–13]. Crizotinib has been shown with inhibitory growth effects on *ROS1*-rearranged NSCLC. In recent clinical studies, patients with advanced NSCLC harboring *ROS1* rearrangements derived great benefit from crizotinib treatment [4,14,15]. However, due to the low frequency of *ROS1* fusion in lung cancers, efficient determination of *ROS1* status in NSCLC patients is critical for directing patient care.

Similar to *ALK* fusion detection, three detection methods, including fluorescence *in situ* hybridization (FISH), immunohistochemistry (IHC), and quantitative real-time reverse transcription-PCR (qRT-PCR), were applied commonly to detect *ROS1* fusion. Each of these methods has their own advantages and disadvantages. Although FISH analysis is considered to be the gold standard method to detect the fusion gene, IHC analysis is technically easy as it is integrated into routine pathological diagnosis. A few studies have demonstrated that IHC is sensitive and specific for determination of *ROS1* status, and is a viable alternative to *ROS1* FISH [12,16–18]. However, due to the low incidence of *ROS1* rearrangement in lung cancer, no study has comprehensively compared these three methods for *ROS1*-fusion detection. In this study, we applied IHC, FISH, and qRT-PCR analysis in a large collection of *ROS1*-positive cases, and compared the specificity and sensitivity of IHC assay with other methods for the detection of *ROS1* fusion in patients with primary lung adenocarcinoma (ADC).

## Materials and Methods

### Clinical materials and tissue microarray (TMA) construction

All included patients had received curative surgery at the Cancer Institute and Hospital, Chinese Academy of Medical Sciences (CICAMS), Beijing, China, between January 2009 and March 2013. All the tumor samples were fixed in 10% neutral buffered formalin for 24–48 h and embedded in paraffin and routinely diagnosed as primary lung ADC. Sixty cases, including 16 ROS1 IHC positive and 44 ROS1 IHC negative cases, were selected from 681 samples, which were previously screened for ROS1 fusion by IHC (data to be published separately). Tissue microarray blocks were built to perform IHC and FISH assays as described previously [19]. This study is retrospective and the data were analyzed anonymously. No images and private information of the patients were released. The Institute Review Board of the Cancer Hospital, CICAMS, agreed to waive the need for consent for this study and approved the study protocol.

### IHC

Immunohistochemical staining was performed on 4 μm-thick TMA slides. Briefly, the slides were deparaffinized and antigen retrieval was then performed in a steam cooker for 1.5 minutes in 1 mM EDTA, pH 9.0 (Maixin Biological Techology Co. Ltd., Fuzhou, China). ROS1 (D4D6) rabbit monoclonal (Cell Signaling Technology, Danvers, MA, USA) was applied at 1:150 in SigalStain antibody diluent (Cell Signaling Technology, Danvers, MA, USA) for 1h. Universal secondary antibody (DAKO) was applied for 15 min. Diaminobenzidine or 3-amino-9-ethylcarbazole was used as chromogens and slides were counterstained with haematoxylin before mounting. ROS1 IHC was scored using the scoring scheme proposed as follows: 0, no staining; 1+, faint cytoplasmic reactivity without any background staining; 2+, moderate cytoplasmic reactivity; and 3+, granular cytoplasmic reactivity of strong intensity in ≥10% of tumor cells.

### FISH

FISH analysis was carried out on 3 μm-thick TMA slides with a break-apart probe specific to the *ROS1* locus (Zyto*Light* SPEC ROS1 Dual Color Break Apart Probe, ZytoVision GmbH, Bremerhaven, Germany) according to the manufacturer’s instructions. Tumor cells, the nuclei of which had one or more FISH signals of each color, were enumerated. The rearrangement-positive cells were defined as those with split signals or isolated green signals. The specimen was considered as *ROS1*-rearranged if the rearrangement-positive cells constituted ≥15% of the enumerated tumor cells.

### QRT-PCR

Total RNA was extracted from 3 to 4 sections of 3 μm-thick FFPE tissue using a RNeasy FFPE kit (Qiagen, Hilden, Germany). The *ROS1* fusion was readily detected by PCR using ROS1 fusion gene detection kit (Amoy Diagnostics Co., Ltd, Xiamen, China) according to manufacturer’s instruction. Briefly total RNA was subject to reverse transcription. Reverse transcription conditions were as follows: 42°C, 1 h; 95°C, 5 min. The resulting complementary DNA (cDNA) solutions were used for a multiplex qRT-PCR. The *ROS1* fusion gene mRNA was detected by qRT-PCR. For each case, 4 reactions were performed to amplify *SLC34A2*-*ROS1*, *SDC*-*ROS1*, *CD74*-*ROS1*, *EZR*-*ROS1*, *TPM3*-*ROS1*, *LRIG3*-*ROS1*, *GOPC*-*ROS1* (Table 1) and the reference gene *HPRT1*. All of the assays were performed on an Agilent Mx3000P QPCR instrument (Agilent Technologies, Santa Clara, CA). The following PCR procedure was used: an initial denaturation at 95°C for 5 min followed by 95°C for 25 s, 64°C for 20 s and 72°C for 20 s to ensure the specificity and 31 cycles of 93°C for 25 s, 60°C for 35 s and 72°C for 20 s to perform the data collection. The quantitative judgment was according to the fusion fluorescence signal. Assay reactions achieving Ct values of <30 cycles were considered positive. A housekeeping gene (*HPRT1*) was used to control the integrity of the RNA.

**Table 1:** The fusion patterns of ROS1 screened in this study.

Reaction	Fusion partners for ROS1, exon	ROS1 exon
1	SLC34A2, 4	32
	SLC34A2, 13del2046	32
	CD74, 6	32
	SDC4, 2	32
	SDC4, 4	32
2	SLC34A2, 4	34
	SLC34A2, 13del2046	34
	CD74, 6	34
	SDC4, 4	34
	EZR, 10	34
3	TPM3, 8	35
	LRIG3, 16	35
	GOPC, 8	35
4	GOPC, 4	36

## Results

### ROS1 IHC

Sixty lung ADCs were included in this study, consisting of 33 female and 27 male patients. The median age is 58 years (range 39–81). Of these, 16 (26.7%, of 60) demonstrated ROS1 protein expression and 44 cases showed no ROS1 expression using IHC analysis. In total of 16 cases with ROS1 protein expression, strong ROS1 expression (3+) was identified in 4 cases, moderate expression (2+) in 8 cases and weak expression (1+) in 4 cases (Table 2).

**Table 2:** Correlation of ROS1 IHC and FISH.

		ROS1 FISH		Total
		Pos	Neg	
IHC	3	4	0	4
				100%
	2	6	2	8
				75%
	1	3	1	4
				75%
	0	0	44	44
				0%
Total		13	47	60
		22%	78%	100%

Pos, positive; Neg, negative.

### *ROS1* FISH and qRT-PCR

**Fig 1:**
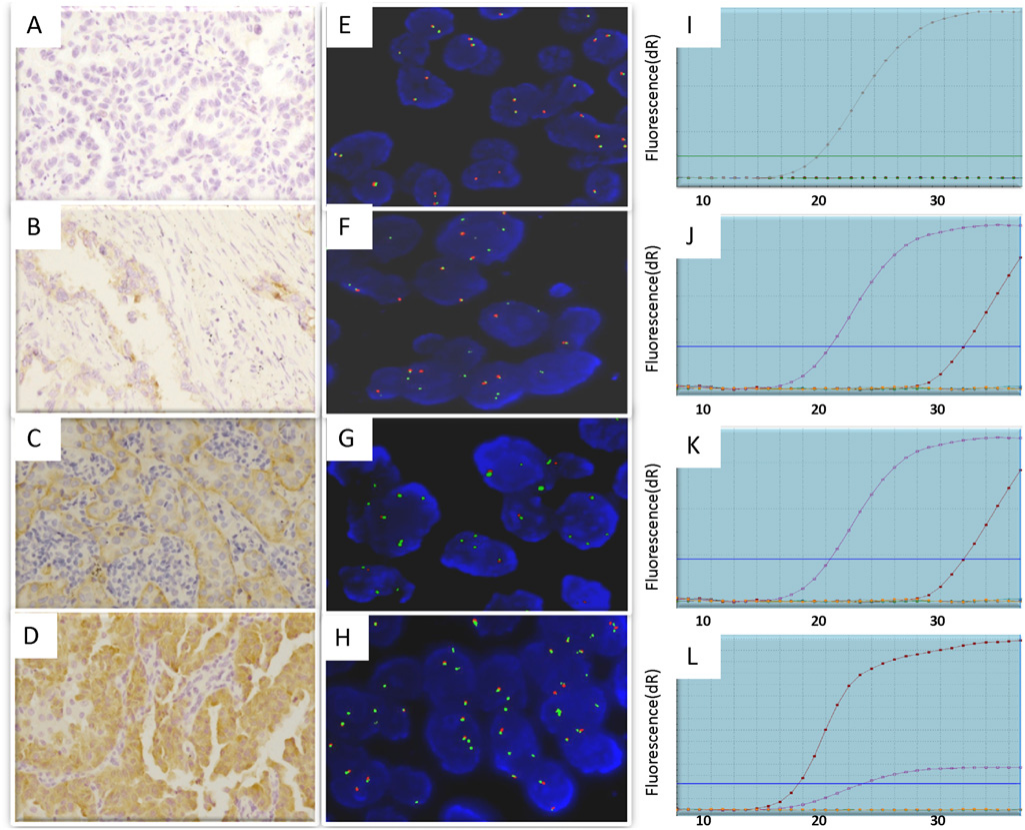
Detection of *ROS1* fusion in lung cancer patients by IHC, FISH and qRT-PCR assays. (A–D) ROS1 IHC staining using D4D6 antibody. (A) Score 0/negative showing no staining; (B) Score 1+ showing faint cytoplasmic reactivity; (C) Score 2+ showing moderate cytoplasmic staining; and (D) Score 3+ showing intense granular cytoplasmic staining in ≥10% of tumor cells. Original magnification ×200. (E–H) FISH analysis using *ROS1* dual color break-Apart FISH probes to detect *ROS1* fusion as split red and green signals. (E) FISH-negative case showing intact two fused signals per nucleus. (F-G) FISH-positive cases representing split (red and green) signals. (H) FISH-positive case showing isolated green signals. Original magnification ×1000. (I–L) Graphs from qRT-PCR showing change in the normalized reporter signal (delta Rn) against PCR cycle number using AmoyDx *ROS1* fusion gene detection kit.

FISH analysis was applied on the 60 lung ADCs, of which 13 cases (21.7%) were identified with an *ROS1* rearrangement (FISH-positive), and 47 cases were not (FISH-negative) (Fig. 1). Split signal pattern was observed in 12 cases and unbalanced rearrangement, characterized by a loss of the red probe, was shown in one case. The qRT—PCR analysis was then performed. Among the 60 cases, 20 cases (33.3%) were identified to be *ROS1*-fusion positive by qRT-PCR analysis (Table 3).

**Table 3:** Correlation of ROS1 qRT-PCR, IHC and FISH.

		IHC		Total			FISH		Total
		Pos	Neg				Pos	Neg	
qRT-PCR	Pos	16	4	20	qRT-PCR	Pos	13	7	20
				33%					33%
	Neg	0	40	40		Neg	0	40	40
				67%					67%
Total		16	44	60	Total		13	47	60
		27%	73%	100%			22%	78%	100%

Pos, positive; Neg, negative.

### Correlation of *ROS1* FISH, IHC and qRT—PCR

As shown in Table 3, of 4 patients with IHC score of 3+, 4 (100%) were FISH positive and all patients with score of 0 (n = 44) were FISH negative. Among the patients with IHC score of 2+ or 1+, 9 (75.0%, 9 of 12) were FISH positive. The sensitivity and specificity of ROS1 IHC with staining intensity score of 1+ or more were 100% and 93.6%, respectively. The sensitivity and specificity of ROS1 IHC with staining intensity score of 2+ or more were 76.9% and 95.7%, respectively.

Thirteen (65%, of 20) patients detected as positive by qRT-PCR were FISH positive, while 40 (100%) patients with *ROS1* negative by qRT-PCR were FISH negative. The sensitivity and specificity of qRT-PCR for detection of *ROS1* fusion were 100% and 85.1%, respectively.

Of 20 *ROS1* qRT-PCR-positive patients, 16 (80%) were IHC positive, while 40 (100%) *ROS1* qRT-PCR-negative cases were IHC negative. The total concordance rate between *ROS1* qRT-PCR and IHC was 93.3%. The 4 discrepant cases for *ROS1* fusion detection between qRT-PCR and IHC analysis were examined using direct sequencing. All the 4 samples were identified with *ROS1* fusion. Two of the four samples have the fusion partner CD74 and another two have the fusion partner EZR. The details of the 20 *ROS1* qRT—PCR-positive cases were shown in (S1 Table).

### Clinicopathological features of *ROS1*-positive patients

In this study, 16 lung ADCs were confirmed with *ROS1*-fusion at least by two methodologies. The *ROS1* fusion-positive cases included 11 women and 5 men with mean age of 56 years (range, 42–75 years). The ratio of never smoker to smokers was 13:3. The predominant histological subtype of *ROS1*-positive patients was acinar in 14/16 (87.5%) of cases, papillary in 10/16 (62.5%), and micropapillary in 8/16 (50.0%).

## Discussion

Application of reliable screening methods for gene rearrangement detection is the key to identify appropriated patients for tumor targeted therapy. In this study, we compared IHC, FISH and qRT-PCR for the detection of *ROS1* fusion in a large cohort of *ROS1*-positive cases together with negative cases.

FISH analysis is often cumbersome and expensive. Moreover it demands fine equipment, skilled personnel, well-preserved FFPE samples, enough cancer cells, etc. For these reasons, it is logistically difficult for many pathology laboratories to support. In contrast, IHC is inexpensive and faster, which is an ideal tool in clinical practice to screen rare but clinically important genetic alterations in tumors. In this study, using FISH as the standard procedure, we demonstrated that the IHC assay with anti-ROS1 (D4D6) Rabbit monoclonal primary antibody is a highly sensitive (100%) and specific (93.6%) method for detection of the *ROS1* rearrangement in primary lung ADC. Although three IHC-positive cases were demonstrated as FISH-negative, their *ROS1* rearrangements were confirmed by qRT-PCR. With the high sensitivity and specificity, ROS1 IHC is a valuable method to rapidly and accurately screen lung cancer patients for appropriate targeted therapy.

Some of the *ROS1*-fusion tumors, which were detected by IHC and/or qRT-PCR, have been reported to show no break-apart FISH signals [17,18,20]. In our study, three cases were demonstrated with negative FISH results, despite repeat testing. Among the three cases, two cases were shown with moderate cytoplasmic staining and one with faint cytoplasmic staining. According to RT-PCR analysis, the partner genes of *ROS1* (6q22) were among SLC34A2 (4q15), CD74 (5q32) and SDC4 (20q12) (Table 1). No matter which gene is the fusion partner, it is on a different chromosome from where *ROS1* locate. Therefore it is theoretically difficult to explain why the split FISH signals could not been seen in these three cases. A possible reason might be that the gene fusion in the three cases occurs at transcription level rather than DNA level. Further study to sequence full length of *ROS1* gene is required to verify this hypothesis. So far no clinical trial has been performed on *ROS1* FISH-negative and IHC-positive NSCLC patients. Therefore it is difficult to predict whether these three patients would be benefit from crizotinib.

QRT–PCR is a fast and sensitive method for detection of expressed known *ROS1* fusion variants for which specific primers have been designed [20]. In this study, the sensitivity and specificity of qRT-PCR for detection of *ROS1* fusion were 100% and 85.1%, respectively. Eleven cases showed mixed *ROS1* fusion patterns. We found that six cases had inconsistent qRT-PCR and FISH results. All the six cases were FISH-negative and qRT-PCR positive, and three of them were IHC-positive. qRT-PCR results were confirmed by direct sequencing of the PCR products. Although sequencing result confirmed the *ROS1* fusion in FISH and IHC negative cases, the fusion were detected in a relatively late stage of the qRT-PCR process (higher *Ct* value). We speculated that the discrepant result from three methods is due to tumor heterogeneity. That is only a small population of cancer cells carry *ROS1* fusion. Another reason might be the *ROS1* fusion occurs in the transcriptional level, but no translation happens.

In conclusion, we have compared IHC, FISH and qRT-PCR for the detection of *ROS1* fusion in a large cohort of *ROS1*-positive cases, and demonstrated that IHC would be served as an effective and rapid detection method in routine pathologic laboratories for the identification of suitable candidates for *ROS1*-targeted therapy. Our study also demonstrated that some ROS1 IHC-positive but FISH-negative lung cancers did harbor the translocation events as confirmed by qRT-PCR. Whether this subgroup of patients would benefit from crizotinib need further clinical trial to provide the evidence.

## Supporting Information

S1 Table List of ROS1-fusion cases detected by qRT-PCR. (DOC)
