# Supplementary material for: Detection of ROS1 Gene Rearrangement in Lung Adenocarcinoma: Comparison of IHC, FISH and Real-Time RT-PCR
Source: PLoS One. 2015 Mar 5;10(3):e0120422. doi: 10.1371/journal.pone.0120422 (PMC4351102; doi:10.1371/journal.pone.0120422)
Supplement: S1 Table — (DOC) [file pone.0120422.s001.doc]

**Table S1. List of ROS1-fusion cases detected by RT-PCR**

| **Sample ID** | **Sex** | **Age** | **Smoking** | **RT-PCR** | **RT-PCR** | **FISH** | **IHC** |
| --- | --- | --- | --- | --- | --- | --- | --- |
|  |  |  | **habit** |  | **Reaction** |  |  |
| 1 | F | 55 | No | Positive | 1 | Negative | 2+ |
| 2 | F | 45 | No | Positive | 1,2 | Positive | 3+ |
| 3 | F | 54 | No | Positive | 1,2 | Positive | 1+ |
| 4 | F | 55 | No | Positive | 1,2 | Positive | 1+ |
| 5 | M | 42 | No | Positive | 1,2 | Positive | 2+ |
| 6 | M | 57 | Yes | Positive | 1 | Negative | 2+ |
| 7 | F | 48 | No | Positive | 1,2 | Positive | 2+ |
| 8 | M | 61 | No | Positive | 2 | Positive | 2+ |
| 9 | M | 75 | Yes | Positive | 1 | Positive | 1+ |
| 10 | F | 66 | No | Positive | 1 | Negative | 1+ |
| 11 | F | 46 | No | Positive | 1,2 | Positive | 2+ |
| 12 | M | 55 | Yes | Positive | 1,2 | Positive | 3+ |
| 13 | F | 43 | No | Positive | 1,2 | Positive | 2+ |
| 14 | F | 57 | No | Positive | 1 | Positive | 3+ |
| 15 | F | 74 | No | Positive | 2 | Positive | 2+ |
| 16 | F | 55 | No | Positive | 1,2 | Positive | 3+ |
| 17 | F | 69 | No | Positive | 1,2 | Negative | 0 |
| 18 | M | 65 | Yes | Positive | 2 | Negative | 0 |
| 19 | M | 49 | Yes | Positive | 1,2 | Negative | 0 |
| 20 | F | 57 | No | Positive | 1 | Negative | 0 |
